# T-Cells Specific for a Self-Peptide of ApoB-100 Exacerbate Aortic Atheroma in Murine Atherosclerosis

**DOI:** 10.3389/fimmu.2017.00095

**Published:** 2017-02-23

**Authors:** Michael K. Shaw, Kevin Y. Tse, Xiaoqing Zhao, Kathryn Welch, Daniel T. Eitzman, Raghavendar R. Thipparthi, Paul C. Montgomery, Ryan Thummel, Harley Y. Tse

**Affiliations:** ^1^Department of Immunology and Microbiology, Wayne State University School of Medicine, Detroit, MI, USA; ^2^Department of Research and Clinical Trials, St. John-Providence Health System, Macomb-Oakland Hospital, Warren, MI, USA; ^3^Division of Rheumatology, Allergy and Immunology, Department of Internal Medicine, University of California at San Diego Medical Center, La Jolla, CA, USA; ^4^Division of Inflammation Biology, La Jolla Institute for Allergy and Immunology, La Jolla, CA, USA; ^5^Cardiovascular Medicine, University of Michigan Cardiovascular Center, Ann Arbor, MI, USA; ^6^Department of Anatomy and Cell Biology, Wayne State University School of Medicine, Detroit, MI, USA; ^7^Cardiovascular Research Institute, Wayne State University School of Medicine, Detroit, MI, USA

**Keywords:** T-cell, atherosclerosis, clones, adoptive transfer, peptides, epitopes

## Abstract

On the basis of mouse I-A^b^-binding motifs, two sequences of the murine apolipoprotein B-100 (mApoB-100), mApoB-100_3501–3515_ (designated P3) and mApoB-100_978–992_ (designated P6), were found to be immunogenic. In this report, we show that P6 is also atherogenic. Immunization of *Apoe*^−/−^ mice fed a high-fat diet (HFD) with P6 resulted in enhanced development of aortic atheroma as compared to control mice immunized with an irrelevant peptide MOG_35–55_ or with complete Freund’s adjuvant alone. Adoptive transfer of lymph node cells from P6-immunized donor mice to recipients fed an HFD caused exacerbated aortic atheromas, correlating P6-primed cells with disease development. Finally, P6-specific T cell clones were generated and adoptive transfer of T cell clones into recipients fed an HFD led to significant increase in aortic plaque coverage when compared to control animals receiving a MOG_35–55_-specific T cell line. Recipient mice not fed an HFD, however, did not exhibit such enhancement, indicating that an inflammatory environment facilitated the atherogenic activity of P6-specific T cells. That P6 is identical to or cross-reacts with a naturally processed peptide of ApoB-100 is evidenced by the ability of P6 to stimulate the proliferation of T cells in the lymph node of mice primed by full-length human ApoB-100. By identifying an atherogenic T cell epitope of ApoB-100 and establishing specific T cell clones, our studies open up new and hitherto unavailable avenues to study the nature of atherogenic T cells and their functions in the atherosclerotic disease process.

## Introduction

Atherosclerosis is a chronic inflammatory condition of medium and large arteries, affecting more than 81 million people in the United States and accounting for 1 in 3 deaths of the resulting cardiovascular disease (1). Traditionally, atherosclerosis is believed to initiate from injuries or dysfunction of endothelial cells lining the inner wall of the vessels (2), thus allowing unabsorbed low-density lipoproteins (LDL) to diffuse into the intima and forming fatty streaks (3). Subsequently, activated macrophages upregulate their endocytic pattern-recognition receptors (4) and internalize oxidized LDL (oxLDL), which gives them their foam-cell appearance (5). These macrophages release inflammatory cytokines and recruit inflammatory immune cells to the site. Continuous influx of mononuclear cells and inefficient clearance of dead cells (efferocytosis) eventually give rise to the formation of atheromatous plaques with a central necrotic lipid core and a fibrous cap (6). Because oxLDL appears to act as an initiator of the disease process (3), current therapies emphasize elevated plasma LDL cholesterol as a major risk factor and statins are prescribed to lower cholesterol levels. However, lowering cholesterol to below 100 or even 70 mg/dl only resulted in a decrease in the number of major adverse cardiovascular events by 30–35% (7). Incomplete prevention suggests that factors other than LDL cholesterol are contributing to the overall atherosclerotic development.

In the last two decades, through the work of Hansson and co-workers (8, 9), it is well established that the immune system, both innate and adaptive, contributes to the progression of the disease (10). First, anti-oxLDL antibodies circulate in patients with atherosclerosis (11). Mice immunized with oxLDL also develop circulating antibodies to the immunogen (12). Second, immune cells are notably found in abundance in human and murine atherosclerotic plaques throughout the stages of disease development (13). Inflammatory T cells and the inflammatory cytokine, interferon-gamma (IFNγ), are found to be determining factors in the development of atherosclerotic plaques. This is supported by the observation that *Apoe*^−/−^ mice deficient in IFNγ or IFNγ receptors exhibit significant reduction in atherosclerotic lesion size compared to controls (14). On the other hand, injection of exogenous IFNγ to *Apoe*^−/−^ mice fed a high-fat diet (HFD) result in a twofold increase in lesion size (15). *Apoe*^−/−^ mice have a defect in the *Apoe* gene (16), which is responsible for transport of chylomicrons and very low-density lipoprotein (VLDL) from the blood stream. *Apoe*^−/−^ mice on chow diet respond to this mutation with high total cholesterol levels >500 mg/dl, mostly in the VLDL and chylomicron remnant fractions. These levels are even more pronounced on an HFD, whereby they develop significant atherosclerosis not seen in an otherwise wild-type mouse. In addition, deficiency of transcription factors or cytokines such as T-bet, IL-12, and IL18 which promote Th1 differentiation and expression of IFNγ genes are shown to reduce atherosclerosis in *Apoe*^−/−^ mice (17). The role of Th17 cells in atherosclerosis, however, has been controversial, as both beneficial and detrimental effects have been reported (18, 19). Evidence that regulatory T cells (Tregs) modulate the disease comes from adoptive transfer studies of CD4^+^CD25^+^ Tregs that resulted in reduction of atherosclerosis in *Apoe*^−/−^ mice (20). Furthermore, a recent human study showed that the number of circulating Tregs in peripheral blood was inversely associated with the development of myocardial infarction and for coronary events in general (21). Thus, a generally projected model is that Th1 cells produce inflammatory cytokines which may be counteracted by Tregs through the production of transforming growth factor beta (22–24). This model, though tenable, was deduced from the observed physical presence of certain T cell subpopulations and their cytokines in the lesions and from gene knockout data. It provides a framework in which many fundamental mechanistic questions are still unresolved. For example, it is unclear how and why T cells are triggered to become atherogenic and what systemic roles they play in the processes of atheroma formation and progression. In addition, while it is assumed that atherogenic T cells belong to the Th1 and Th17 subclasses, isolation of a homogenous population of such atherogenic T cells has not been successful. We contend that these questions require new strategies as well as novel experimental approaches to fully understand the mechanisms of T cell atherogenicity. In this respect, we adopt a basic immunological approach and aim to unravel the events that initiate the T cell atherogenic response. Since T cells are triggered to respond mostly through their antigen receptors, we reason that identification of the atherogenic peptides would provide a direct pathway leading to isolation and characterization of the atherogenic T cells themselves.

We previously described identification of two immunogenic T cell epitopes, mApoB-100_3501–3515_ (designated P3) and mApoB-100_978–992_ (designated P6), derived from the apolipoprotein B-100 (ApoB-100) of LDL. Immunization of C57Bl/6, *Apoe*^−/−^, or *Ldlr*^−/−^ mice with P3 or P6 emulsified in complete Freund’s adjuvant (CFA) induced strong peptide-specific T cell proliferative responses *in vitro* (25). In this report, we provide further evidence that P6 is an atherogenic epitope. Immunization of *Apoe*^−/−^ mice with P6 emulsified in CFA resulted in a significant increase of aortic atherosclerotic lesions in mice fed a high-fat Western diet. More importantly, exacerbation of aortic atheroma development can be adoptively transferred to unprimed recipients with P6-primed T cells as well as P6-specific T cell clones. This new experimental model allows us, for the first time, to obtain homogeneous populations of antigen-specific atherogenic T cells for analysis. It also opens up new possibilities to study the nature of atherogenic T cell populations and their functional significance in the disease process.

## Animals and Methods

### Mice

Female C57BL/6 mice were purchased from the Jackson Laboratory (Bar Harbor, ME, USA). Male and female *Apoe*^−/−^ mice on the C57BL/6 background were bred in our colonies. In some experiments, mice were fed a HFD starting at 8 weeks of age. All experimental procedures involving live animals received prior approval from Institutional Animal Care and Use Committee of Wayne State University.

### Antigens and Peptides

All murine ApoB-100 (mApoB-100) peptides and MOG_35–55_ peptides were synthesized by Genemed Synthesis Inc (San Antonio, TX, USA). Peptides were dissolved in phosphate-buffered saline (PBS) and adjusted to 2 mg/ml. Human apolipoprotein B-100 (hApoB-100) full-length protein was purchased from Abcam (Cambridge, MA, USA) and was dialyzed extensively against PBS before use.

### Identification of mApoB-100 IA^b^-Binding Motifs

Based on analysis of the alignment of 64 peptides that bound to IA^b^, Liu et al. (26) delineated a set of possible residues that most frequently serve as anchor residues for IA^b^ binding. In this analysis, four major histocompatibility complex (MHC) contact positions were defined (peptide positions 1, 4, 6, and 9) and the three most common amino acids used at each site were listed (position 1, A, F, and Y; position 4, S, A, and P; position 6, V, P, and A; position 9, S, V, and A). This analysis also provided potential T cell interacting residues in positions 2, 3, 5, and 8. We arbitrarily deemed any 9-mer peptide with three MHC contact residues to be potential I-A^b^-binding peptides. We developed a strategy to scan for such binding motifs along the 4505 amino acid sequence of ApoB-100. We first searched for any of the three amino acids found in MHC contact position 1 (A, F, or Y) and designated this amino acid position 1 of our potential 9-mer. We then determined the amino acids corresponding to positions 4, 6, and 9 downstream of the first position. If any two of the remaining three positions also had an amino acid used frequently in I-A^b^-binding peptides, we designated the 9-mer as a candidate for further characterization. Similarly, we searched the ApoB-100 sequence for amino acids corresponding to those used frequently in position 4. We then searched upstream and downstream from this residue for amino acids corresponding to positions 1, 6, and 9. This strategy defined another subset of potential 9-mers. We repeated this search starting with amino acids often used in positions 6 and 9 of the I-A^b^-binding motif. Two more subsets of potential 9-mers were thus defined. In all, 18 potential I-A^b^-binding 9-mers were designated. Given their primary sequence, six of these were determined to have adequate water solubility to be used in experiments.

### Immunization of Mice

For immunization, protein antigens and peptides at 2 mg/ml were emulsified with an equal volume of CFA supplemented with 1 mg/ml of *Mycobacterium tuberculosis* H37Ra (Difco Laboratories, Detroit, MI, USA). Then, 0.05 ml of the emulsion was given s.c. at each of four sites in the flanks draining the inguinal and axillary lymph nodes (27). Mice were used 10–14 days after immunization.

### Cell Cultures and Adoptive Transfer

For bulk cultures (27), the inguinal and axillary lymph nodes were removed aseptically 10 days after immunization and teased into single cell suspensions. Cells were washed twice and put into culture at 4 × 10^6^/ml in RPMI supplemented with 10% fetal calf serum, 2 mM l-glutamine, 1 mM MEM sodium pyruvate, 0.1 mM MEM non-essential amino acids, 100 U/ml penicillin, 100 μg/ml streptomycin, 10 mM HEPES buffer, and 5 × l0^−5^ M 2-ME together with the specific antigens. Cells were cultured in 2-ml volume in 24-well plates at 37°C with 5% CO_2_/air for 4 or 5 days as specified.

For adoptive transfer, cells were harvested, washed, and resuspended in PBS to appropriate volumes. Each recipient received 0.2 ml of a cell suspension containing 5 × 10^7^ viable cells through the tail veins.

### T Cell Proliferative Assay (28)

Draining lymph nodes were harvested and teased into single cell suspensions 10–14 days after immunization. A total of 3 × 10^5^ viable cells per microtiter well were cultured with 50–100 μg/ml of the peptides for 4 days. In some cases, purified protein derivative (PPD) served as a positive control, and a peptide of MOG or OVA served as negative controls. Sixteen hours before harvesting, 1 μCi of tritiated thymidine was added to each well. Cells were harvested and incorporation of ^3^H was determined in a scintillation counter. Results are expressed as stimulation indexes (SIs) using the following formula:

$$\text{SI}=\frac{\left(\text{counts per minute (cpm) experimental}-\text{cpm medium control}\right)}{\text{cpm medium control}}.$$

### Atherosclerosis Quantification

The procedures used were adopted from the Eitzman Lab (29). *Apoe*^−/−^ mice were perfused with saline at physiological pressure and then fixed using formalin with a 25-gauge needle inserted into the left ventricle at a rate of 1 ml/min. The arterial tree was then meticulously dissected and placed in 70% ethanol. After staining with Oil Red O, the aorta was pinned on wax. Pinned aortic tissue was imaged using a SPOT camera (Spot Imaging; Sterling Heights, MI, USA) mounted to a Leica M165FC Stereomicroscope (Leica Biosystems; Nussloch, Germany). Identical settings were used for all samples. The surface area occupied by atherosclerotic plaques was quantified at the aortic arch and major branches with Image-Pro Plus software (Media Cybernetics, Bethesda, MD, USA). The lesion area was expressed as a percentage of total surface area examined.

### T Cell Cloning

T cells were cloned by the limiting dilution method of MacDonald et al. (30, 31). Briefly, lymph node cells from *Apoe*^−/−^ mice primed 10–12 days previously with P6/CFA were placed in bulk cultures in 2-ml volumes with P6 at 25 μg/ml in 24-well plates. After 1 week in culture, half of the media in each well was replaced with fresh media plus 25 U/ml of mouse recombinant IL-2 without fresh antigen or antigen-presenting cells. After 1 week, cells were harvested, washed, and resuspended to concentrations containing 30,000, 3,000, 300, and 30 cells/well, respectively. Then, 0.1 ml of each cell concentration was dispensed to each well of a 96-round-bottom-well plate. Total cell concentration in each well was brought up to 3 × 10^5^ by addition of appropriate numbers of irradiated (3,000 rad) syngeneic spleen cells. P6 was used at 50 μg/ml and mouse recombinant IL-2 at 25 U/ml. Signs of T cell growth in the round-bottomed wells as a ring of elongated cells surrounding the cell pellet were determined by visual inspection of individual wells on an inverted microscope. Plates with less than 37% positive wells with growth were considered clonal according to the Poisson distribution theory (31). Wells with positive growth went through 2-week cycles of stimulation and resting as described above for bulk cultures. Positive wells were expanded to 24-well plates and then T15 culture flasks. Testing of antigen specificity was performed with T cell proliferative assays.

## Results

### Immunogenic T Cell Epitopes of Apo-B100 Serve as a Lead to Identify Atherogenic T Cells

**Figure 1:**
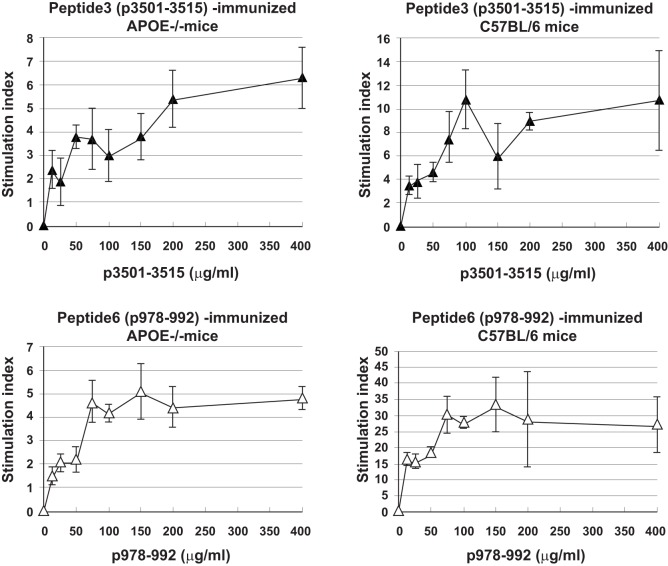
**Titration curves of T cell proliferative responses to peptide3 (ApoB-100p3501–3516) and peptide6 (ApoB-100p978–993)**. C57BL/6 or *Apoe*^−/−^ mice were immunized with P3 or P6. Ten days later, draining lymph nodes were isolated and teased into single cell suspension. A total of 2 × 10^5^ lymph node cells were cultured with no or titrating concentrations of their respective priming peptides for 5 days. Sixteen hours before the termination of the cultures, 1 μCi of tritiated thymidine was added to each well. Cultures were harvested in an automatic cell harvester, and the incorporation of ^3^H was counted in a liquid scintillation counter.

Immune T cells are triggered most commonly through the T cell receptor (TCR) by binding of a ligand that is composed of an antigenic peptide embedded within an MHC molecule. It is thus reasonable to expect that elucidation of a defined peptide that is atherogenic would provide the means for identification and isolation of atherogenic T cell populations useful for functional studies under controlled conditions. To this end, we made use of the concept of the H-2 IA^b^-binding motifs described by Liu et al. (26) to search for IA^b^-binding peptide sequences of mApoB-100, an apolipoprotein molecule found on the surface of the LDL particle (32). Each LDL particle has a single copy of ApoB-100 (33). We identified 18 sequences that fit the IA^b^-binding motifs, 6 of which were synthesized for further study. These six 15-mer-peptides were designated peptides 1–6 (25). *Apoe*^−/−^ mice were immunized with each peptide followed by an extensive analysis of the responses to the respective peptide and to each other as measured in a T cell proliferative assay (28). PPD and concanavalin A (Con A) were used as positive controls. Lymph node cells of mice immunized with P3 (mApoB-100_3501–3515_) or P6 (mApoB-100_978–992_) mounted strong T cell proliferative responses to their respective peptides (Table 1). No apparent cross-reactivity was detected between the two peptides. Titration of antigen doses in B6 and *Apoe*^−/−^ mice to P3 and P6 also showed a dose dependency of the T cell proliferative responses (Figure 1). By contrast, P1, P2, P4, and P5 were not immunogenic and did not elicit any positive T cell responses in immunized mice. These results were confirmed by physical binding affinity data in which P3 and P6 were shown to have high-binding affinities to I-A^b^, while the other four peptides had low-binding affinities (34). The observed responses to ApoB-100 peptides were not confined to *Apoe*^−/−^ mice only. C57BL/6 and *Ldlr*^−/−^ mice immunized with these peptides had similar response patterns (25). In addition, the proliferative responses induced by P3 and P6 immunization were largely due to CD4^+^ T cells. As shown in Table 2, P3- or P6-primed *ApoE*^−/−^ lymph node cells positively selected for CD4^+^ or CD8^+^ T cells by antibody-coated microbead columns (Miltenyi Biotec) were assessed in T cell proliferative assays. The bulk of the lymph node cell proliferative responses were confined to the CD4^+^ T cell population for both peptides, while the CD8^+^ T cell population did not appear to contribute to the overall responses. These results indicated that P3 and P6 sequences are immunogenic and are able to invoke an immune response through activation of CD4^+^ T cells.

**Table 1:** **T cell proliferative responses of *Apoe*^−/−^ mice to apolipoprotein B-100 (ApoB-100) peptides**.

Immunization with ApoB-100 peptides	T cell proliferative responses [counts per minute and stimulation index (SI)]^a^								
	Medium	Peptides for *in vitro* stimulation (μg/ml)							
		P1 (50)	P2 (50)	P3 (50)	P4 (50)	P5 (50)	P6 (50)	Purified protein derivative (PPD) (20)	Con A (1)
P1 (mApoB-100_3066–3080_)	2631 ± 187	3,524 ± 209 (0.34)	3,904 ± 341 (0.48)	4,791 ± 528 (0.82)	4,763 ± 406 (0.81)	3,136 ± 255 (0.19)	2,508 ± 341 (−0.06)	19,697 ± 1,571 (6.49)	15,603 ± 2,089 (4.93)
P2 (mApoB-100_438–452_)	1,998 ± 164	1,807 ± 365 (−0.10)	1,244 ± 173 (−0.38)	2,689 ± 404 (0.35)	4,197 ± 273 (1.10)	3,411 ± 557 (0.71)	3,049 ± 586 (0.53)	15,486 ± 1,623 (6.79)	24,273 ± 2,907 (11.15)
P3 (mApoB-100_3501–3515_)	1,728 ± 226	2,447 ± 253 (0.42)	1,695 ± 208 (−0.02)	**13,064 ± 1,507 (6.56)**	1,918 ± 202 (0.11)	1,594 ± 183 (−0.09)	2,067 ± 204 (0.20)	10,981 ± 1,334 (5.35)	15,543 ± 1,828 (7.99)
P4 (ApoB-100_1578–1592_)	3,039 ± 415	2,188 ± 407 (−0.28)	4,637 ± 298 (0.53)	1,906 ± 204 (−0.12)	2,385 ± 175 (−0.22)	3,563 ± 420 (0.17)	2,917 ± 483 (−0.04)	20,089 ± 1,472 (5.61)	18,146 ± 1,469 (4.97)
P5 (ApoB-100_4054–4068_)	1,386 ± 277	2,845 ± 639 (1.05)	3,247 ± 303 (1.34)	2,055 ± 148 (0.48)	2,162 ± 257 (0.60)	1,849 ± 103 (0.33)	2,408 ± 376 (0.74)	18,924 ± 2,681 (12.65)	16,901 ± 2,011 (11.19)
P6 (ApoB-100_978–992_)	3,236 ± 496	4,805 ± 611 (0.49)	2,735 ± 591 (−0.15)	3,804 ± 681 (0.18)	4,539 ± 509 (0.40)	5,001 ± 786 (0.55)	**28,723 ± 4,162 (7.88)**	22,508 ± 2,937 (5.96)	25,924 ± 2,062 (7.01)

*Separate groups of *Apoe*^−/−^ mice were immunized in four sites in the flanks with each of the ApoB-100 peptides emulsified in complete Freund’s adjuvant. Ten days later, popliteal and axillary lymph node cells were isolated. The cells were cultured separately with or without 50 μg/ml of each of the peptides for 4 days. PPD and concanavalin A (Con A) were included as positive control. Sixteen hours before the end of the culture period, 1 *μ*Ci of tritiated thymidine (^3^H) was added to each well. Cells were harvested on an automatic cell harvester and incorporation of ^3^H was determined in a Beckman scintillation counter. All cultures were established in triplicates, and the results were expressed as the mean number of counts per minute ± SEM*.

^a^SIs are calculated by the following formula: $\frac{\mathrm{counts per minutes (CPM) experimental}-\mathrm{counts per minute (CPM) medium background}}{\mathrm{counts per minute (CPM) medium background}}.$ *Bold font indicates the important data*.

**Table 2:** **CD4^+^ and CD8^+^ T cell subsets responding to peptides 3 and 6**.

			*In vitro* proliferative responses (stimulation index)^a^		
Immunizing Ag (200 μg/mouse)	Cell populations (2 × 10^5^ cells)	Medium (cpm)	Peptide3 (100 μg/ml)	Peptide6 (100 μg/ml)	MOG_35–55_ (100 μg/ml)
Peptide 3	Unseparated	2,387	**18.0 ± 4.6**	0.0 ± 0.5	−1.0 ± 0.2
	CD4^+^	1,911	**24.0 ± 6.0**	0.0 ± 0.3	−1.0 ± 0.1
	CD8^+^	1,764	−1.0 ± 0.0	−0.1 ± 0.0	−1.0 ± 0.3
Peptide 6	Unseparated	2,005	0.0 ± 0.4	**9.0 ± 0.8**	−1.0 ± 0.2
	CD4^+^	1,793	0.0 ± 0.4	**22.0 ± 3.2**	−1.0 ± 0.1
	CD8^+^	1,862	0.0 ± 1.9	0.0 ± 0.3	2.0 ± 0.5

*Apoe^−/−^ mice were immunized with P3 or P6 as described in Table 1. Ten days later, draining lymph node cells from both groups were isolated. The cells were passed through anti-CD4 microbead MAC columns and anti-CD8 columns from Miltenyi Biotech to positively isolate CD4^+^ and CD8^+^ T cells, respectively. Purified cells, together with unseparated cells were assayed for T cell proliferative responses. All cultures were established in triplicates, and the results were expressed as the mean number of counts per minute ± SEM*.

^a^SIs are calculated by the following formula: $\frac{\mathrm{counts per minutes (CPM) experimental}-\mathrm{counts per minute (CPM) medium background}}{\mathrm{counts per minute (CPM) medium background}}.$ *Bold font indicates the important data*.

### Only P6 (mApoB-100_978–992_) Is Both Immunogenic and Atherogenic

**Figure 2:**
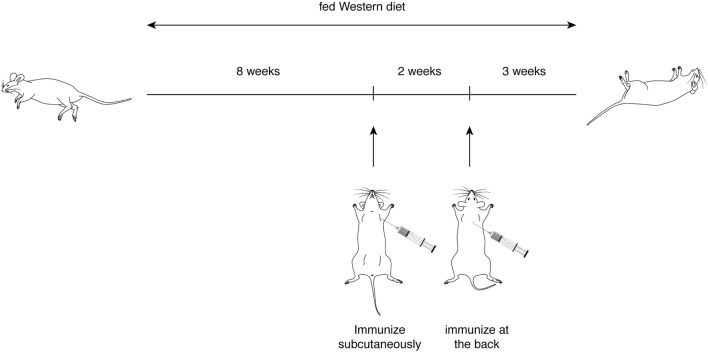
**Experimental scheme for immunization of *ApoE*^−/−^ mice with apolipoprotein B-100 peptides**.

**Figure 3:**
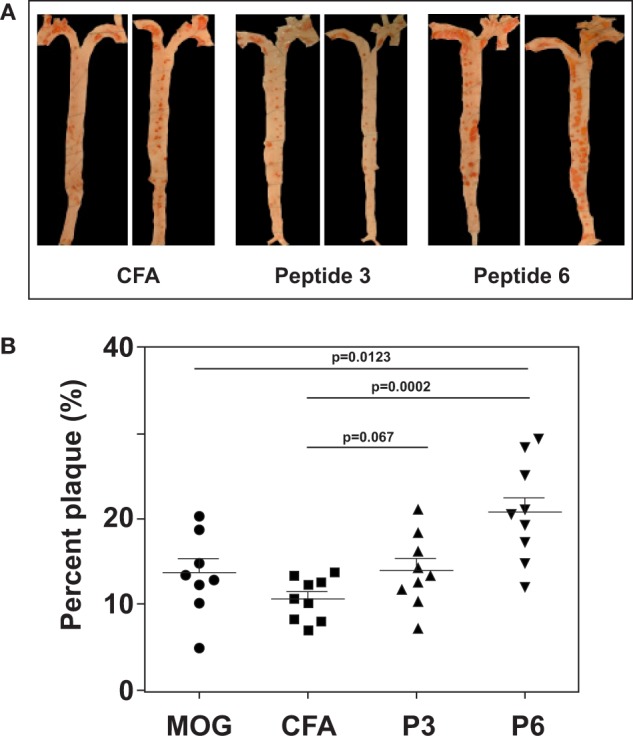
**Immunization with P6 enhanced development of atherosclerosis in Western diet-fed *Apoe*^−/−^ mice**. *Apoe*^−/−^ mice were fed a high-fat diet for 5 weeks. Groups of mice were then immunized with P3, P6, MOG_35–55_, or saline emulsified with complete Freund’s adjuvant by procedures described in Table 1. Two weeks later, mice were given a boost with 100 μg of the same priming peptide subcutaneously at the back. Three weeks later, aortas were isolated according to the procedures developed in the Eitzman Lab (29). After staining with Oil Red O, the aorta was pinned on wax. Pinned aortic tissue was imaged, and the surface area occupied by atherosclerotic plaques was quantified at the aortic arch and major branches with Image-Pro Plus software. The lesion area was expressed as a percentage of total surface area examined. **(A)** Representative aortas from each group. **(B)** Dot plot of data.

With the first identification of two unique T cell epitopes of ApoB-100, we next determined whether these epitopes were also atherogenic. The experimental design depicted in Figure 2 was followed. In this experiment, all groups of mice were fed a HFD for the 10-week duration. The mice were immunized after 5 weeks of feeding. MOG_35–55_, a peptide of myelin oligodendrocyte glycoprotein, served as an irrelevant peptide control. Since all peptide antigens were emulsified in CFA and CFA itself may be inflammatory, a second control with saline emulsified in CFA was used. At the end of 10 weeks, mice were sacrificed, and the aortic roots and aortic arches were isolated following the methods used by Eitzman and co-workers (29). The extent of plaque coverage was assessed *via* en face staining of the aortic samples with Oil Red O. Figure 3 shows that mice immunized with saline emulsified in CFA and fed an HFD provided a baseline level of plaque area coverage for comparison with other groups. Mice immunized with MOG_35–55_ emulsified in CFA had similar background plaque area level, indicating that this irrelevant peptide had no effect on atherosclerotic plaque burden as a result of the HFD. In contrast, mice immunized with P6 emulsified in CFA and with an HFD had greatly increased total area of plaque coverage, thus demonstrating the atherogenic nature of P6. This increase in plaque coverage was highly significant when compared to saline–CFA and MOG_35–55_–CFA controls. Surprisingly, mice immunized with P3, under similar conditions, did not show such an enhancement of disease. We conclude that while some ApoB-100 peptides may be immunogenic, they are not necessarily also atherogenic. The basis of the differences in atherogenicity between P3 and P6 is not known at this point but is under investigation. Nevertheless, the fact that P6 peptides are atherogenic offers a novel experimental model to study the mechanisms of T cell epitope-specific atherogenic development from an immunological perspective.

### Adoptive Transfer of P6-Primed Lymph Node T Cells Exacerbates Atherosclerosis

**Figure 4:**
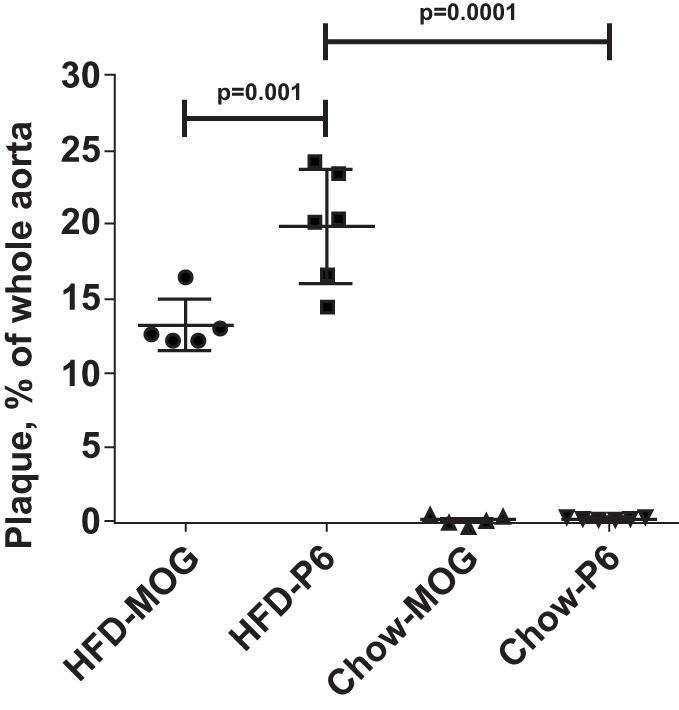
**High-fat diet feeding was necessary for enhanced atherosclerotic lesions**. *Apoe*^−/−^ mice were fed a high-fat diet or regular mouse chow. Five weeks later, the two groups of mice received 5 × 10^7^ lymph node cells from donor *Apoe*^−/−^ mice immunized with either P6 or MOG_35–55_ peptides. The primed donor lymph node cells were cultured with their respective antigens for 5 days before adoptive transfer. The HFD-P6 and HFD-MOG groups continued to be fed a HFD for another 5 weeks. The chow-P6 and chow-MOG groups were fed regular mouse chow. After 5 weeks, mouse aortas were isolated and stained with Oil Red O, and the areas of atherosclerotic plaques were quantified. The lesion area was expressed as a percentage of total surface area examined.

The previous experiments established an association between immunization with P6 and exacerbation of atherosclerotic development. To demonstrate direct causal effects of P6 priming and disease exacerbation, we performed adoptive transfer of P6-primed lymph node cells into unprimed recipients and assessed the effects on development of atherosclerotic plaques. *Apoe*^−/−^ mice were immunized with P6 or MOG_35–55_ peptides emulsified in CFA. Ten days later, draining lymph node cells from each group were harvested and cultured in bulk with the respective antigens for 5 days. At the end of the culture period, 5 × 10^7^ viable cells were adoptively transferred through the tail vein into 12-week-old *ApoE*^−/−^ recipients which had been fed a HFD for 5 weeks prior to receiving cell transfer and were continued to be fed the HFD for the next 5 weeks post cell transfer. Mice fed a HFD and received P6-primed lymph node cells exhibited enhanced atherosclerotic plaque burden when compared to those in control mice receiving the irrelevant antigen MOG_35–55_-primed lymph node cells (Figure 4). These data unequivocally demonstrate a causal role of P6-specific T cells in aggravating plaque formation in atherosclerosis.

### P6-Specific T Cells Exacerbate but Do Not Initiate Atherosclerosis Development

To reveal whether the pre-feeding with HFD was necessary to manifest the enhancement effects of P6 immunization, we also adoptively transferred P6-primed and MOG-primed lymph node cells into two separate groups of 12-week-old *Apoe*^−/−^ recipients which were fed the regular mouse chow throughout rather than given the HFD. As demonstrated in Figure 4, *Apoe*^−/−^ mice fed regular chow and receiving either MOG-primed or P6-primed lymph node cells did not exhibit any aortic lesions, suggesting that an inflammatory environment created by feeding HFD is a critical factor for the atherosclerosis-promoting function of P6-specific T cells. Relevant to this conjecture, we have demonstrated in a separate study (Wolf et al., submitted for publication) that feeding *Apoe*^−/−^ mice an HFD led to rapid loss of P6/IA^b^ tetramer^+^Foxp3^+^ Tregs.

### P6 Is a Naturally Processed Peptide of ApoB-100

We further determined if P6 can mimic a naturally processed physiologic peptide of the native ApoB-100 molecule. We immunized *Apoe*^−/−^ mice with the native ApoB-100 molecule and tested *in vitro* if P6 could stimulate a proliferative response in ApoB-100-primed lymph node cells. As it is not realistically possible to extract sufficient amount of mouse ApoB-100 protein from mouse sera for testing, we purchased full-length hApoB-100 protein from a commercial source (Abcam, Cambridge, MA, USA). The P6 peptide sequence N′-TGAYSNASSTESASY-C′ (mApoB-100_978–992_) of mouse ApoB-100 is 100% homologous to its counterpart in hApoB-100. Table 3 shows that lymph node cells from mice immunized with full-length hApoB-100 molecule responded to stimulation *in vitro* with not only the priming antigen hApoB-100, but also with P6. Similarly, lymph node cells from mice immunized with P6 responded to the priming antigen P6 as well as the hApoB-100 full-length protein. Since hApoB-100-primed T cells recognized and responded to P6 stimulation, this is indicative that a peptide identical to or cross-reactive with P6 was generated in the antigen processing of hApoB-100. Indeed, in support of this conjecture, in a separate collaborative study, Wolf et al. (submitted for publication) showed that P6/IA^b^ tetramer^+^ P6-specific T cells were present in normal *Apoe*^−/−^ mice.

**Table 3:** **T cell proliferative responses of human apolipoprotein B-100 (hApoB-100)-primed lymph node cells**.

		*In vitro* proliferative responses [stimulation index (SI)]^a^			
Immunizing Ag (100 μg/mouse)	Medium (cpm)	hApoB-100 (50 μg/ml)	P6 (50 μg/ml)	Purified protein derivative (PPD) (20 μg/ml)	OVA (100 μg/ml)
hApoB-100	3,354	3.30	4.03	8.35	0.04
P6	3,567	2.86	7.25	8.87	0.15

*Groups of Apoe^−/−^ mice were immunized with hApoB-100 native molecule and P6 following the procedures described in Table 1. T cell proliferative assay was performed 10 days later. PPD was used as positive controls and OVA as negative controls. All cultures were established in triplicates*.

^a^SIs are calculated by the following formula: $\frac{\mathrm{counts per minutes (CPM) experimental}-\mathrm{counts per minute (CPM) medium background}}{\mathrm{counts per minute (CPM) medium background}}.$

### P6-Specific T Cells Clones Exacerbate Atherosclerosis in Recipient Mice

To gain further understanding of the nature of the atherogenic T cells, we generated P6-specific T cell clones by limiting dilution (30, 31). Nine P6-specific T cell clones were initially derived and validated by T cell proliferative assay. Many of these clones had SIs of 80–150 (Table 4). Clones P6-11 was uniformly IFNγ producing as analyzed by flow cytometry (Table 4), which was conceivable as the clones were originally derived from mice immunized with CFA, which is T_H_1 polarizing. All T cell clones were recloned to assure clonality, aliquoted, stored frozen, and subsequently tested for antigen specificity and atherogenicity.

**Table 4:** **Proliferative responses of P6-specific T cell clones**.

	*In vitro* proliferative responses					% of cloned T cells secreting interferon gamma (IFNγ) assessed by flow cytometry intracellular staining	
Clone # (5 × 10^4^ cells)	MediumCPM	Peptide 6		Purified protein derivative (PPD)		T cell clones stimulated with	
		CPM	Stimulation index (SI)	CPM	SI	PPD	Peptide 6
P6-6	17	47	1.8				
P6-9	27	289	2.8				
**P6-11**	27	1,562	**56.2**	164	1.1	3.31	80.25
**P6-12**	27	2,231	**80.8**	314	10.6		
**P6-13**	24	964	**39.2**				
**P6-14**	26	1,205	**45.3**				
**P6-15**	15	426	**26.8**				
**P6-24**	14	2,090	**148.3**				
P6-25	37	65	0.8				

*5 × 10^4^ P6-specific T cell clones were cultured with or without 50 μg/ml of P6 together with 5 × 10^4^ irradiated spleen cells as APC in 96-well culture plates. Some cultures contained 20 μg/ml of PPD as negative control. Sixteen hours before termination of cultures, 1 *μ*Ci of tritiated thymidine (^3^H) was added to each well. The results were expressed as mean counts per minute and as stimulation indexes. The clonality of clone P6-11 was assessed by intracellular staining for IFN*γ*. P6-11 was stimulated in culture for 48 h with either PPD or Peptide 6 and 10^6^ irradiated spleen cells as antigen-presenting cells, some of which might have survived the 48-h culture and appeared as non-IFN*γ*-secreting cells*.

*Bold font indicates the important data*.

**Figure 5:**
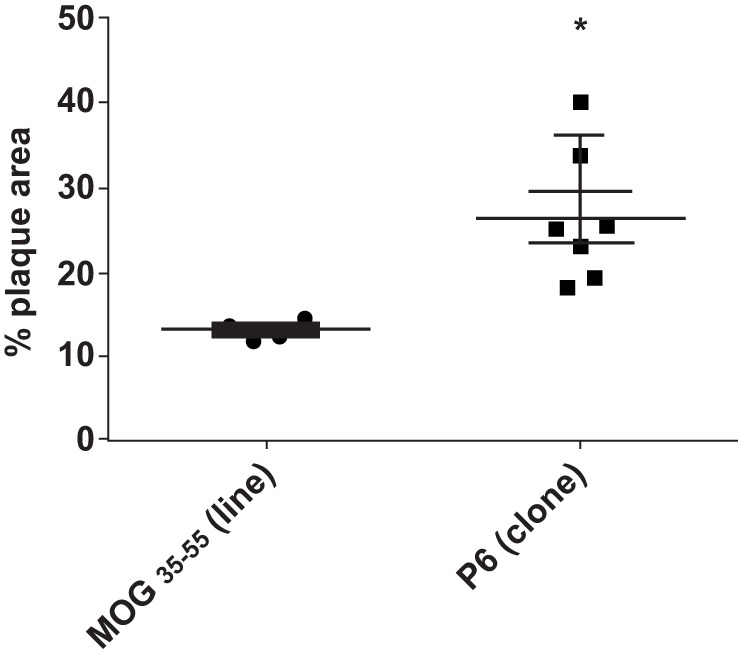
**P6-specific T cell clone adoptively transferred exacerbation of atherosclerotic development T cell clones were derived by the limiting dilution technique (31)**. *Apoe*^−/−^ mice were fed a high-fat diet for 5 weeks. The mice were divided into two groups. A total of 2 × 10^7^ T cell clone specific for P6 or T cell line specific for MOG_35–55_ were adoptively transferred into each group. Five weeks later, the mice were analyzed for the extent of plaque lesions *via* en face staining of the aortic samples with Oil Red O. *p = 0.001.

To assure that the cloned T cells maintained their atherogenicity, we adoptively transferred through the tail vein 2 × 10^6^ P6-11 T cell clones or 2 × 10^7^ MOG_35–55_-primed cell line, which had been maintained for several cycles (not cloned), into 12-week-old *Apoe*^−/−^ mice which had been fed a HFD for 5 weeks prior to receiving the cells. After additional 5 weeks of feeding post cell transfer, the extent of plaque lesions was assessed *via* en face staining of the aortic samples with Oil Red O. Mice that received P6-11 cloned cells had greatly enhanced atherosclerotic development as compared to mice that received the MOG_35–55_-primed control cells (Figure 5). These results indicate our ability to obtain a homogenous population of atherogenic T cells. These T cell clones offer a new opportunity to further understand the role of T cells in the development of atherosclerotic lesions.

## Discussion

This report describes a new strategic approach establishing an experimental system to study T cell atherogencity. First, we identify a self-peptide mApoB-100_978–992_ (designated P6) of mouse ApoB-100, which is a T cell epitope and is clearly involved in the development of atherosclerotic lesions. Second, T cells specific for this peptide have been cloned and are shown to adoptively transfer exacerbation of plaque development in recipients. It is also interesting to observe that while these T cell clones exacerbate the disease, they do not seem to initiate the development of atherosclerosis. To our knowledge, this is the first study that identifies a T cell epitope and generates epitope-specific T cell clones which are atherogenic. A recent report published in 2012 by Wick’s group (35) described the proliferative responses of T cells generated from human atherosclerotic lesions by stimulation with peptides of heat shock protein 60 (HSP60). These proliferative T cells presumably were atherogenic because they were generated from atherosclerotic lesions, but this presumption was not directly demonstrated. A similar study with T cell clones derived from human atherosclerotic plaques were reported by Benagiano et al. (36) in 2005. These T cell clones mounted strong proliferative responses to hHSP60 peptides, but their atherogenicity was only indirectly implied (see below). Another study by Stemme et al. (37) derived T cell clones from human atherosclerotic plaques using polyclonal mitogens as stimuli and found that these T cell clones responded to oxLDL in proliferative responses and secretion of cytokines. The specific peptides of oxLDL that stimulated the clones were not elucidated. In our case, the adoptive transfer of P6-primed T cells and P6-specific T cell clones in Figures 3 and 4, respectively, clearly correlates these cells with the exacerbation of atherosclerotic development. The availability of the P6-specific T cell clones is an advance that will allow further understanding of the nature of the atherogenic T cells and the mechanisms of how they are triggered. In addition, the feeding of HFD in combination with immunization with P6 also greatly speed up the development of full-blown atherosclerosis to 10 weeks rather than the conventional 25–30 weeks by HFD alone.

Our approach to elucidate the antigenic peptides of ApoB-100 that bind to and stimulate peptide-specific T cells, as a means to identify and isolate atherogenic T cells, was based on the work of Liu et al. (26). The Kappler and Marrack laboratory analyzed the binding properties of 64 peptide sequences to murine I-A^b^ and derived a set of I-A^b^-binding motifs, which predict the possible MHC-binding anchor residues and hence the sequences of peptides that have the physical and chemical properties of fitting into the antigen-binding cleft of I-A^b^. Since *Apoe*^−/−^ mice are also of the H-2^b^ haplotype, their analysis provided us a unique opportunity to identify possible atherogenic peptides of mApoB-100. Subsequently, the observation that P6, but not P3, is atherogenic, even though both peptides can induce strong T cell proliferative responses (Table 1), is interesting. This investigation is ongoing.

Previous work studying ApoB-100 peptides came from Nilsson and co-workers. P210 (peptide 210) is one of over 100 hApoB-100 peptides initially identified through their recognition by circulating autoantibodies found in patients with atherosclerotic lesions (38, 39). It was found that the presence of high concentrations of circulating autoantibodies specific for P210 corresponded to less risk of atherosclerosis development and *vice versa*. In animals immunized with ApoB-100 peptides, atherosclerosis was reduced by 40% (40). The mechanisms of how immunization with P210 results in atheroprotection, however, are not fully elucidated. CD8^+^ T cells, Th2, and Tregs are all implicated in the process (41–43). It must be pointed out that there are differences in the use of P210 verses P6. First, P210 was identified through binding to autoantibodies and is supposed to be a B-cell epitope of ApoB-100. On the other hand, P6 was identified based on I-A^b^-binding motifs to T cells and is supposed to be a T cell epitope. Second, immunization with P210 requires conjugation to a carrier BSA and is injected with the adjuvant alum (aluminum hydroxide) (40). For P6, the peptide is emulsified directly with the adjuvant CFA. Alum and incomplete Freund’s adjuvant are known to induce suppressive Th2 and Treg responses (44, 45), while CFA tends to induce inflammatory Th1 responses (46). Overall, it seems that the mechanisms of atheroprotection are more complex than anticipated. On the other hand, by first understanding the basis of the atherogenicity of P6, we expect that the process of identifying antigen-specific therapeutic targets for atherosclerosis can be more direct. We are also mindful that P6 is only one of the 18 ApoB-100 peptides screened by the I-A^b^-binding motif approach. There may be other atherogenic determinants affecting the development of atherosclerosis.

This study also uniquely identifies P6 as a physiologic product of antigen processing. In mice immunized with ApoB-100, the protein is processed by APC into a set of peptides to be presented to T cells. T cells with appropriate TCRs specific for some immunogenic peptides undergo clonal expansion and increase in frequencies to detectable levels. The observation that P6 was able to stimulate a T cell proliferative response in lymph node cells from ApoB-100-immunized mice, but not MOG_35–55_-immunized mice (Table 3), indicated that P6-responsive T cells were among those T cells that had expanded. We conclude that in the pool of naturally processed peptides of Apo-B100, one must be identical to or cross-reactive with P6. Indeed, this conclusion is supported by the work of Wolf et al. (manuscript submitted) in separate experiments using P6/I-A^b^ multimers to track P6-specific T cells showing that at 4 weeks old, *Apoe*^−/−^ mice already harbored a high frequency of P6-specific T cells in their mesenteric lymph nodes.

The current study lends credence to the concept that atherosclerosis is an autoimmune disease, although the initial proposal for autoimmune atherosclerosis used HSP60 as the model (47, 48). With the identification of P6 as a self-T cell epitope, and the demonstration of P6-specific T cells as direct cause of exacerbating atherosclerosis, the two basic criteria for the definition of an autoimmune disease are corroborated. In this scenario, we note that the mere presence of atherogenic P6-specific T cells does not lead to induction of atherosclerosis; but in the event of a local inflammation, these cells would act to aggravate the inflammatory condition, resulting in exacerbation of the atherosclerotic lesions (Figure 4). We attribute these results to the need of the immune system to break tolerance to a self-antigen, and inflammation provides the stimuli for higher expression of co-stimulatory molecules to facilitate antigen presentation. These results are also consistent with the correlation that patients with autoimmune diseases tend to have a higher risk of also developing cardiovascular disease (49).

Collectively, the identification of a T cell atherogenic epitope of ApoB-100 opens up new avenues whereby the mechanism of T cell functions in the development of atherosclerosis can be more closely studied. For example, the availability of P6/I-A^b^ tetramers already helped demonstrate the presence of P6-specific T cells in normal 4-week-old *Apoe*^−/−^ mice (Wolf et al., manuscript submitted). In addition, it is also anticipated that the construction of a P6-specific TCR transgenic mouse can be used to study the ontology and the evolution of T cell atherogenesis. More importantly, attempts can be made to reprogram the pathologic P6-specific T cell clones into induced pluripotent stem cells (50, 51), to be followed by their redifferentiation into functional Tregs maintaining the same P6 specificity. This type of individualized adoptive immunotherapy should be favored over global inhibition of the immune system as a means to treat autoimmune diseases (52, 53).

## Author Contributions

All the authors assisted in data collection and analysis, and assisted in manuscript preparation and revision. MS, KT, KW, XZ, and HT designed the experiments. DE and RT provided reagents, equipment, and technical support. MS, KT, XZ, KW, and RT performed the experiments. PM and HT wrote the manuscript.

## Conflict of Interest Statement

The authors declare that the research was conducted in the absence of any commercial or financial relationships that could be construed as a potential conflict of interest.

## Abbreviations

HFD, high-fat diet; HEL, hen egg lysozyme; *Ldlr*^−/−^, low-density lipoprotein receptor deficient; PPD, purified protein derivative.
